# On Gall-Stones and Their Treatment

**Published:** 1893-03

**Authors:** 


*On Gall-Stones and their Treatment.* By A. W. MAYO ROBSON, F.R.C.S. Pp. x., 285. London: CASSELL & COMPANY, LIMITED. 1892.

This little work is the outcome of a very considerable experience in the surgery of the gall-bladder, and, in a small compass, presents us with an outline of the best modern method of dealing with a class of diseases which were formerly the exclusive domain of the physician, but are coming more and more into the hands of the surgeon.

The opening chapters are devoted to anatomy and physiology, and are amplified by a series of observations on the secretion of bile in a case of biliary fistula, published by the author in the *Proceedings of the Royal Society*. These observations showed that the average quantity of bile which flowed in 24 hours was about 30 ounces, and that more was invariably excreted by day than by night. Some of the results obtained were in direct antagonism to views once held by physiologists: thus, the antiseptic properties of bile were found to be unimportant, the supposed stimulating effect of bile on the intestinal walls was not necessary for a regular action of the bowels, and the excretion of bile was not materially influenced by diet nor by cholagogue drugs, such as calomel, euonymin, and podophyllin.

Succeeding chapters are devoted to the pathology, symptoms, and diagnosis of gall-stones; but the best part of the book is that which treats of the four modern operations known as cholecystotomy, cholelithotrity, cholecystectomy, and cholecystenterostomy. These are illustrated by notes of 41 operations done by the author, with only four deaths, three of which were in cases of malignant disease.

Mr. Mayo Robson prefers cholecystotomy to cholecystectomy, and would limit the latter operation to the following circumstances:

(1) Where the gall-bladder is contracted, and cannot be sutured to the parietes.

(2) Where the walls of the gall-bladder are so changed by ulceration as to make suture impossible.

(3) Where a fistula persists after the milder operation.

These restrictions, which are no doubt good, would limit the more radical operation to very small dimensions.

The author apologises in the preface for reporting his cases with too much detail; but in our opinion this part of the book is by far the most valuable, giving evidence of the perfection to which operations upon abdominal organs have been brought by Mr. Mayo Robson and others, and showing how much interference may be borne with impunity by organs like the gall-bladder, which were formerly thought to be out of reach of surgical treatment.

We can recommend the book as a good and trustworthy guide to our present knowledge of the treatment of gall-stones.

